# Resilience: Now What? Comment on "Re-evaluating Our Knowledge of Health System Resilience During COVID-19: Lessons From the First Two Years of the Pandemic"

**DOI:** 10.34172/ijhpm.8563

**Published:** 2024-08-06

**Authors:** Laura Kihlström, Soila Karreinen

**Affiliations:** ^1^Welfare State Research and Reform, Finnish Institute for Health and Welfare, Helsinki, Finland; ^2^Health Sciences Unit, Faculty of Social Sciences, Tampere University, Tampere, Finland

**Keywords:** Health System Resilience, Health policy, Preparedness, COVID-19, Transformation, Crisis

## Abstract

In this paper we draw upon the review article "Re-evaluating Our Knowledge of Health System Resilience During COVID-19: Lessons from the First Two Years of the Pandemic" by Saulnier et al to propose some additional themes to be considered regarding ongoing conversations on health system resilience. Complementing the lessons learned brought forward in the article, we propose three thematic areas which may enrichen this conversation. These three themes are posed as questions: (1) Transformation – towards what? (2) Crises and shocks – what counts as such? and (3) Levels and scales – can tensions be reconciled? While our insights are strongly rooted in research results on health system resilience during COVID-19 in Finland, we seek to discuss their wider implications for health system resilience and beyond the context of a single country

## Health System Resilience and COVID-19

 The article by Saulnier et al draws together literature on health system resilience during the first two years of COVID-19, with the intent that studying health system shocks and responses to them can “enhance our understanding of health system resilience and establish a clearer link between theoretical concepts and practical ideas on how to build resilience.”^1^ The article is a narrative literature review, largely based on the health system resilience framework introduced by Blanchet et al in 2017,^2^ exploring the prevailing academic understanding(s) of dimensions and uncertainties regarding health system resilience. The authors conclude that while more research is needed on several sub-topics and components of health system resilience (eg, teamwork, actor legitimacy, values, and inclusivity), the findings show “the utility of resilience theory for strengthening health systems for crises.” Saulnier et al acknowledge that their analysis “presents a review of resilience through one lens” and that “it would be worthwhile to conduct further analyses using other frameworks” “to generate a more comprehensive assessment of where the concept of health system resilience currently stands.”^1^

 We have written our comment as a response to this call. Our commentary is not based on a single resilience framework per se, rather we build it upon emerging literature on health system resilience since the publication of Saulnier and colleagues’ article. Given the breadth of this literature, we claim in no way to be exhaustive in our remarks, rather, we bring up themes and questions to further enrich and refine existing resilience theory, building on our own research completed on health system resilience, COVID-19 and crises.^3-6^ We have structured our commentary under three main subtopics posed as questions, each containing issues to be further addressed by literature on health system resilience.

## Transformation – Towards What?

 Saulnier et al bring forward that literature on health system resilience tends to focus more on adaptation and absorption, with less attention given to the transformative capacity of health systems.^1^ Transformation suggests a marked change in form, function, and in ways of doing things while absorption and adaptation can be described as restoring and modifying activities.^2^ Absorption and adaptation are usually detected in earlier stages of a shock and typically require less reflexivity. The authors point out that the “tendency to equate resilience with maintaining essential health services or with emergency preparedness may also draw attention away from possible structural and functional changes and towards short-term change in particular sub-systems or areas.”^1^ We argue that understanding the reasons for scant engagement with health system transformation requires a deeper engagement with the epistemological foundations of the study of health systems more generally.

 The “what” of transformation may be too easily avoided in health system resilience research not only due to temporal aspects, such as the time period of studying shocks and crises, but also due to a tendency of health system resilience research to replace the ingrained political aspects of health systems with techno-managerial and professional vocabulary.^6-8^ As a result, value-based discussions – which are at the core of transformation – regarding health systems are often left to the realm of politics.^7^ What the COVID-19 pandemic has shown, however, is that value-based discussions, ethical considerations, and visions for health systems can *also* be elusive or non-existent in the realm of politics, and instead decisions may often be justified with techno-managerial language and metrics. In fact, resilience of the system may also be used as a technical justification for decisions which have inequitable outcomes.^4,6,9-10^

 We also find it important for health system resilience researchers to critically assess the temporality and comprehensiveness of transformation. For an action to be considered as transformation, does it only include permanent, fundamental change at the system level, or does it also include more ephemeral forms of transformation? During the COVID-19 pandemic many health systems took on modes of management, service delivery and collaboration that can be perceived as transformative (besides being adaptive), which may have been abandoned since the first two years of the pandemic. One example is using other workforce than healthcare professionals within healthcare organizations. Such cross-boundary recruitment includes fundamental change in thinking and acting but was reversed after the pandemic.^3^ In other words, assessing transformation should also differentiate between system-level transformation as well as smaller scale transformation, particularly for countries in which health systems are highly decentralized.

## Crises and Shocks – What Counts as Such?

 Saulnier et al write that “resilient health systems have the capacity to absorb shocks using existing resources while maintaining the same essential functions as before, adapt to them by adjusting their functions and use of resources, or fundamentally transform their functions to reduce risks in response to the shock.” In other words, health system resilience literature places an emphasis on shocks, shock absorbance, shock recovery, reactions to crises, and strengthening of health systems for future shocks. Critical takes on the usage(s) of the crises concept have emphasized that the term “reduces our analysis of a particular situation to the query, “What went wrong?,” which then presents deviations or errors as “aberrations of the normal operation of things.”^11^ Therefore, a resilience paradigm focused on shocks and crises can be critiqued from the perspective that by emphasizing adaptability and abilities to cope with crises, it “naturalizes” and even renders invisible the preconditions, root causes and global interconnections which create such shocks and crises in the first place.^12^

 In other words, crises and shocks are not natural phenomena but intimately social, impacted by the broader political, cultural, and social contexts in which they take place.^13^ This notion could shift the attention of resilience literature to how crises and shocks affect different communities – at the local and global level – unequally, and how such differing vulnerabilities are created. Such a shift would highlight that resilient health systems should not exist solely for periods of crises, and that resilience “must not amount to the temporary alleviation of the symptoms of a more profound socially created vulnerability.”^12^

## Levels and Scales – Can Tensions Be Reconciled?

 During COVID-19, both research and media have strengthened the framing of health systems as nationalsystems. During the first two years of the pandemic, it was commonplace to read reports about successful (and unsuccessful) pandemic responses, which often ranked countries in terms of a narrow set of indicators.^14^ Such metrics and a focus on success within a single country in one sector (ie, intensive care) might render invisible the rundown of other sectors (ie, primary care, elective care). Also, different regions have very different performances and starting points. In our research, we found that the local level of the health system was often side-tracked from decision-making processes and even left outside political decision-making.^3,5,6^ Therefore, the critical question to ask is how resilience frameworks and theories may reproduce this framing by focusing on “system components” rather than the diversity, ie, localized capacities and strengths which may enhance health system resilience. This tension between generalization and specificity is, of course, a challenge for any theoretical framework. Health system resilience literature could be enriched by studying topics such as governance and leadership also at the local level, which is often the face for crises for most citizens.

 Some countries have started taking steps towards strengthening resilience capacities through “resilience testing,” which involves collaboration across health system levels.^15^ However, investigating and identifying localized capacities and strengths requires also reflexivity regarding research approaches and methodologies used in health system resilience research. Much of our current understandings of health system resilience during crises, such as COVID-19, come from elite sources, such as government documents and health system leaders, which do not sufficiently portray how resilience is shaped, built, weakened, or maintained among communities most affected by crises and shocks.

## Conclusion

 Saulnier and colleagues’ article focuses on lessons learned during the first two years of the pandemic, which is also a period during which COVID-19 probably became one of the most researched pandemics in history. As Saulnier et al point out, while the pandemic is no longer considered an acute crisis, now is the time to continue taking stock of what happened, how, and why, and what the long-term implications of the pandemic have been on health systems and resilience.^1^ In this comment, we have sought to highlight some of the tensions, assumptions, and unaddressed issues in the results brought forward by Saulnier et al, with the intention of contributing to an ongoing refinement of resilience theory in health systems and policy research.

## Ethical issues

 Not applicable.

## Competing interests

 Authors declare that they have no competing interests.
